# Supplementary figures and images for: Micro-scale control of oligodendrocyte morphology and myelination by the intellectual disability-linked protein acyltransferase ZDHHC9
Source: eLife. 2025 Oct 1;13:RP97151. doi: 10.7554/eLife.97151 (PMC12488188; doi:10.7554/eLife.97151)

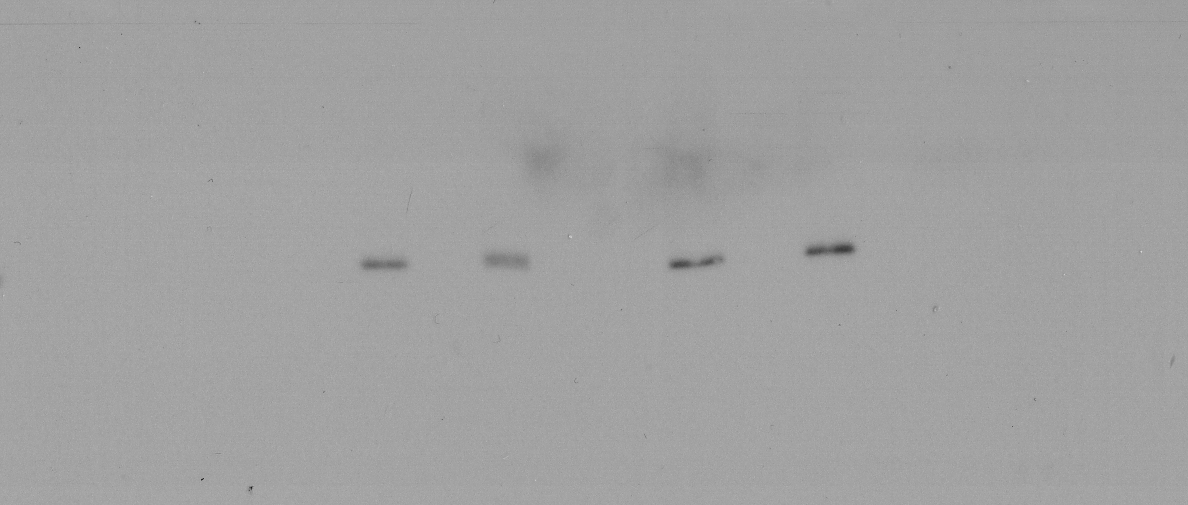

Supplement: Figure 8—source data 1. [file elife-97151-fig8-data1.zip › HA-ZDHHC9 Inputs ABE Fig 8A.tif]

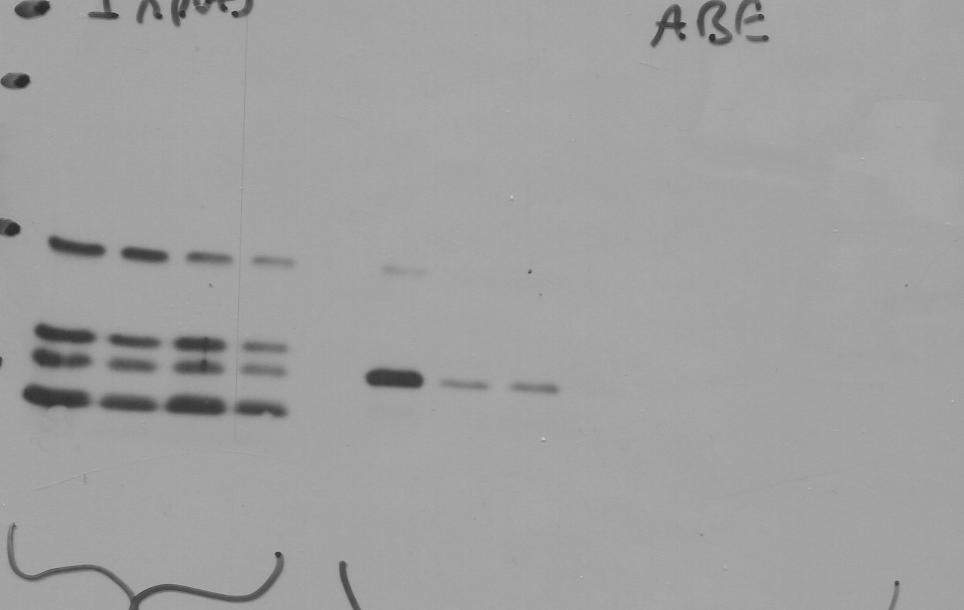

Supplement: Figure 8—source data 1. [file elife-97151-fig8-data1.zip › MBP Inputs and ABE in vivo Fig 8C.tif]

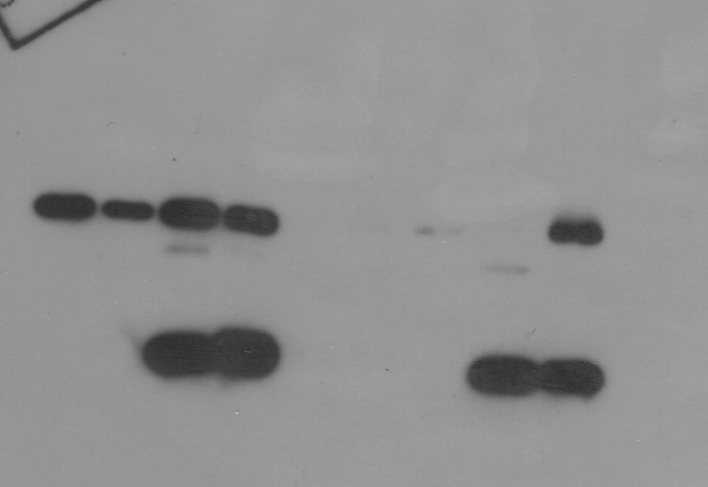

Supplement: Figure 8—source data 1. [file elife-97151-fig8-data1.zip › Myc (MBP Golga-7) Inputs ABE Fig 8A.tif]

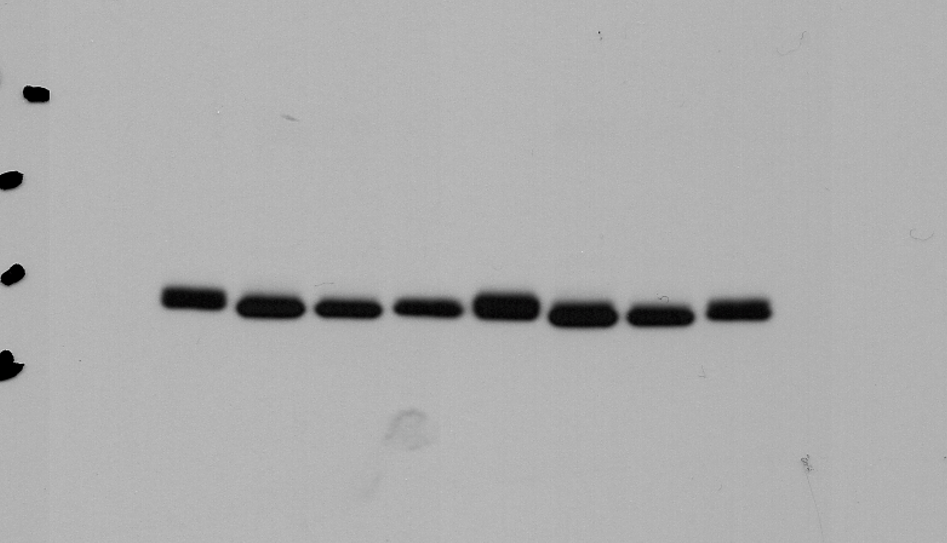

Supplement: Figure 8—source data 1. [file elife-97151-fig8-data1.zip › Fig 8E ZDHHC9 Input.tif]

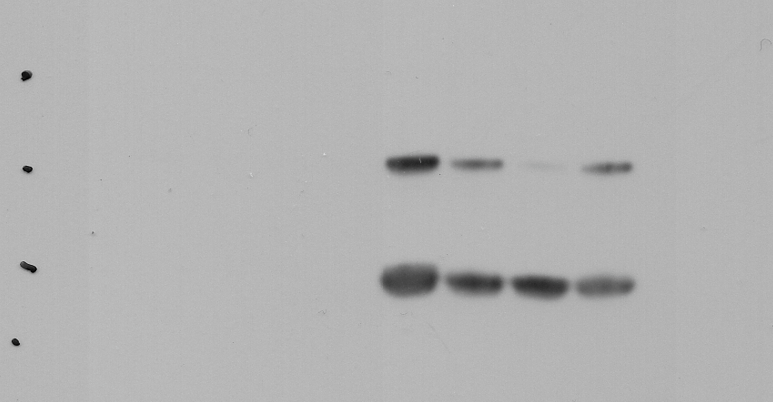

Supplement: Figure 8—source data 1. [file elife-97151-fig8-data1.zip › Fig 8E MBP Golga7 ABE.tif]

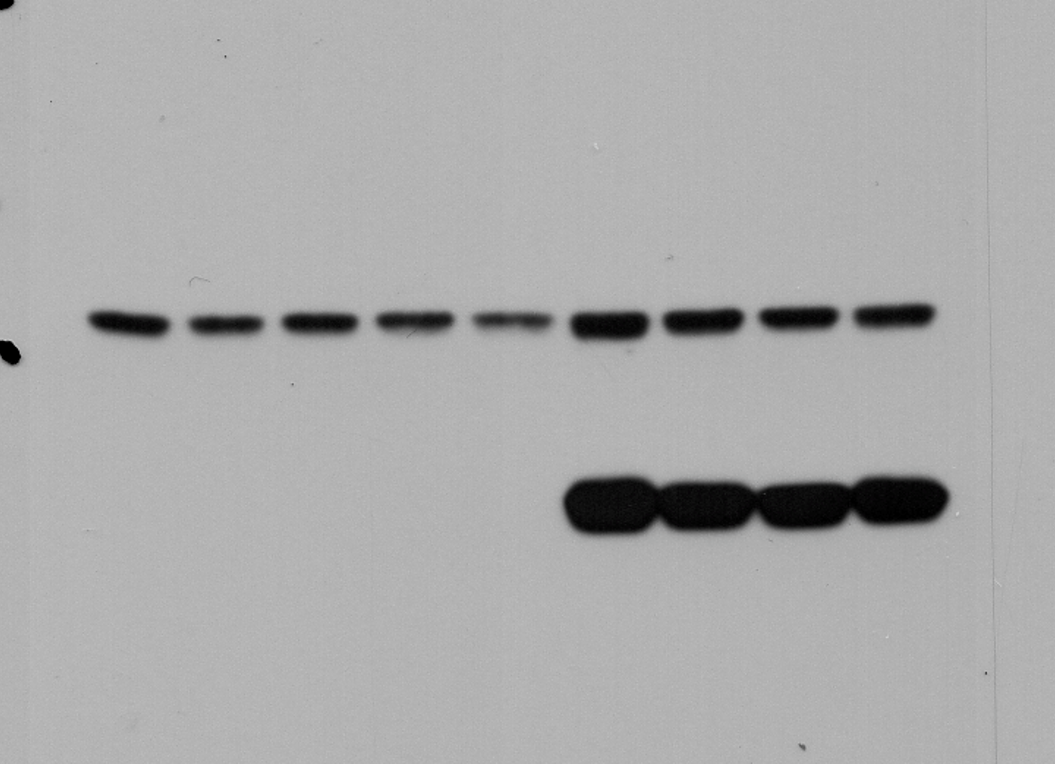

Supplement: Figure 8—source data 1. [file elife-97151-fig8-data1.zip › Fig 8E MBP Golga7 input.tif]

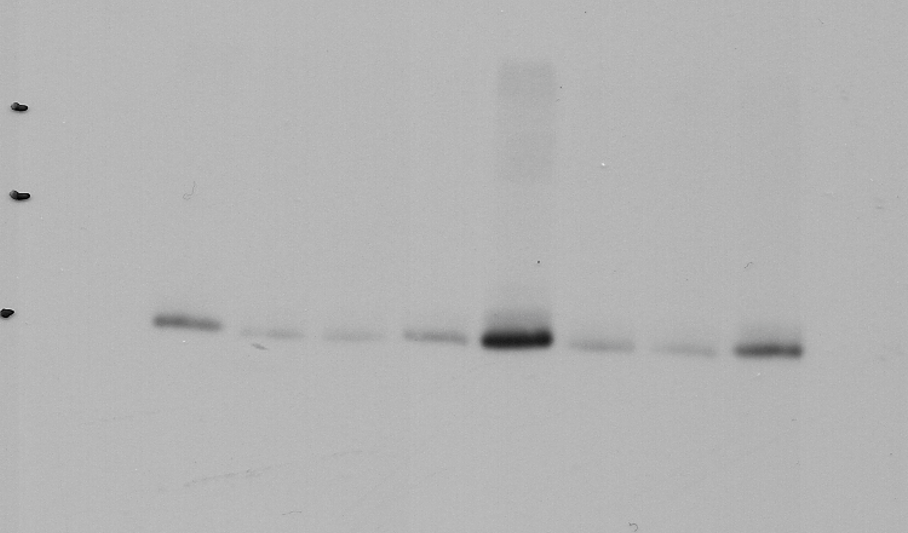

Supplement: Figure 8—source data 1. [file elife-97151-fig8-data1.zip › Fig 8E ZDHHC9 ABE.tif]

|   |   |   |   |   |   |   |   |   |                    |
|---|---|---|---|---|---|---|---|---|--------------------|
| - | + | - | + | - | + | - | + | + | HA-ZDHHC9          |
| - | - | + | + | - | - | + | + | + | Myc-Golga7         |
| + | + | + | + | + | + | + | + | - | NH <sub>2</sub> OH |

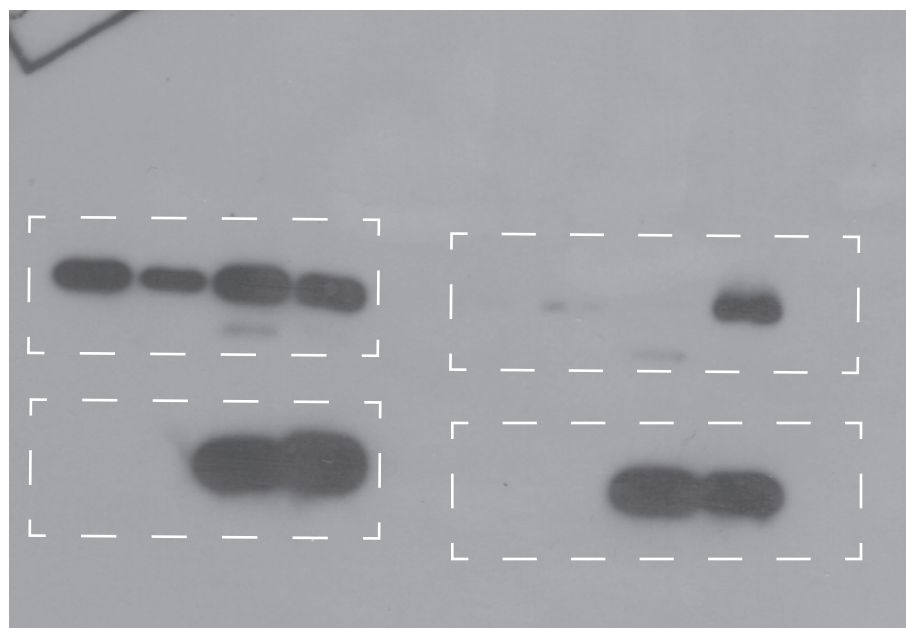

|   |   |   |   |   |   |   |   |   |                    |
|---|---|---|---|---|---|---|---|---|--------------------|
| - | + | - | + | - | + | - | + | + | HA-ZDHHC9          |
| - | - | + | + | - | - | + | + | + | Myc-Golga7         |
| + | + | + | + | + | + | + | + | - | NH <sub>2</sub> OH |

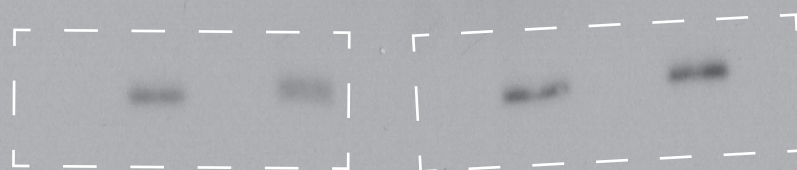

Supplement: Figure 8—source data 2. [file elife-97151-fig8-data2.pdf]

wt ZDHHC9 KO

wt ZDHHC9 KO

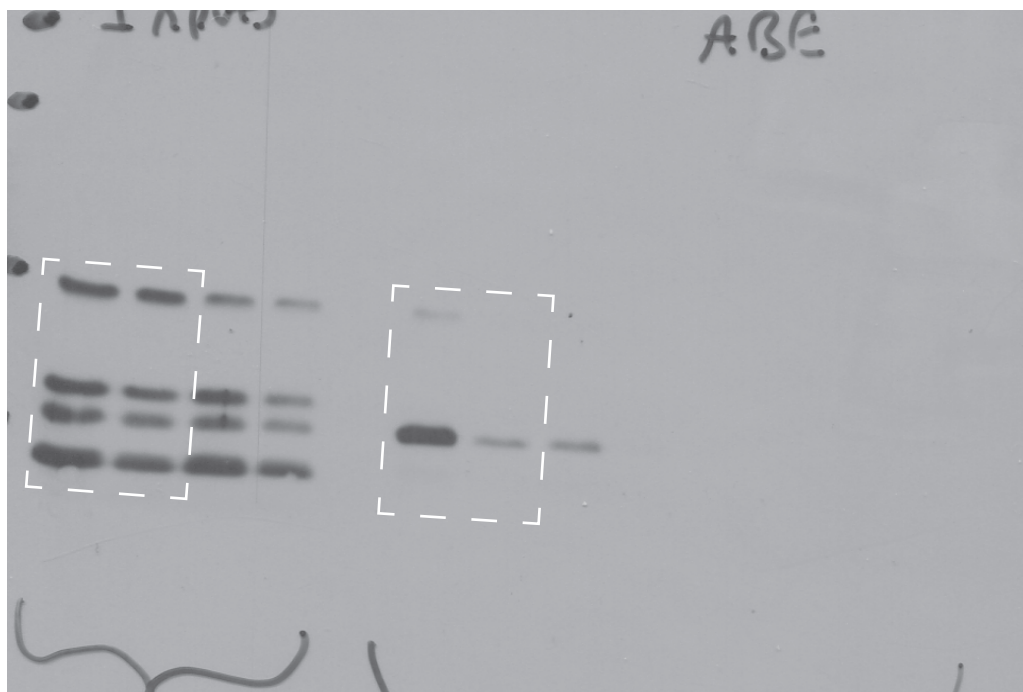

Supplement: Figure 8—source data 3. [file elife-97151-fig8-data3.pdf]

|   |    |      |       |       |    |      |       |       |    |                    |
|---|----|------|-------|-------|----|------|-------|-------|----|--------------------|
| - | wt | R96W | R148W | P150S | wt | R96W | R148W | P150S | wt | HA-ZDHHC9          |
| - | -  | -    | -     | -     | +  | +    | +     | +     | +  | Myc-Golga7         |
| + | +  | +    | +     | +     | +  | +    | +     | +     | -  | NH <sub>2</sub> OH |

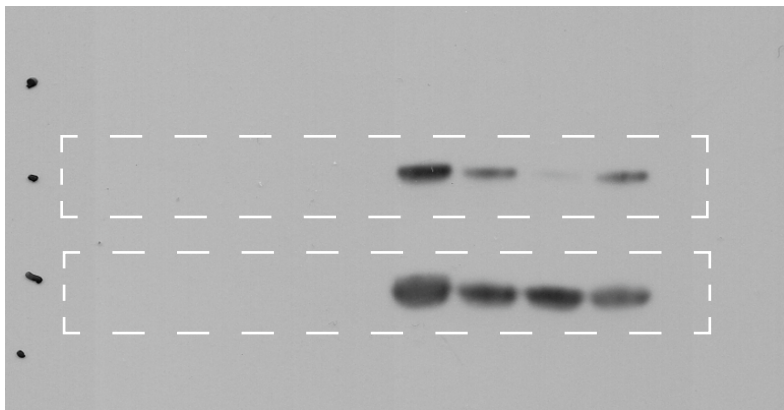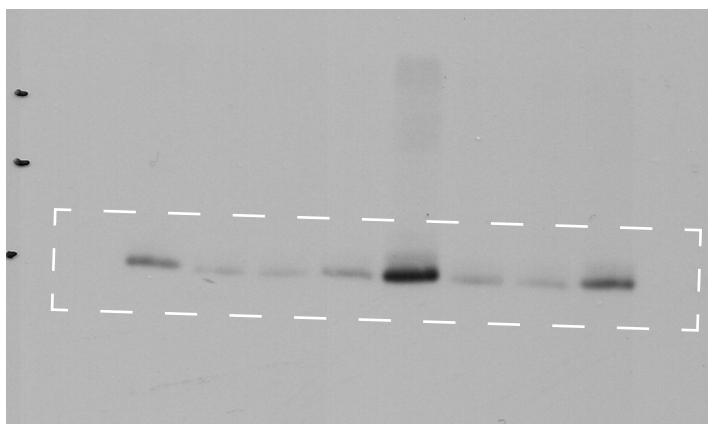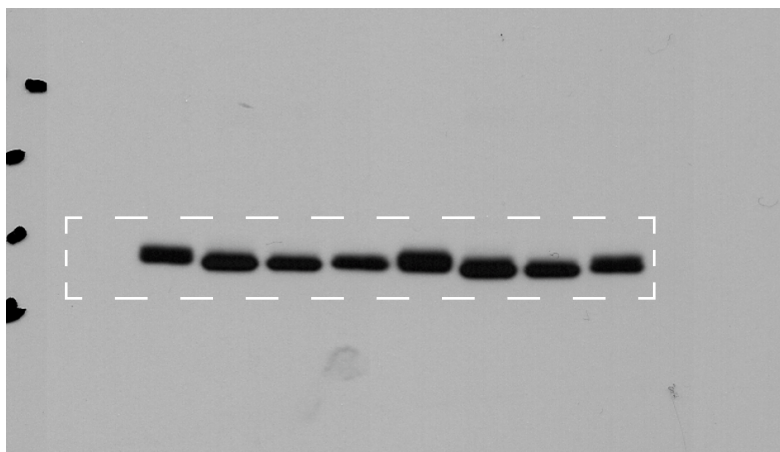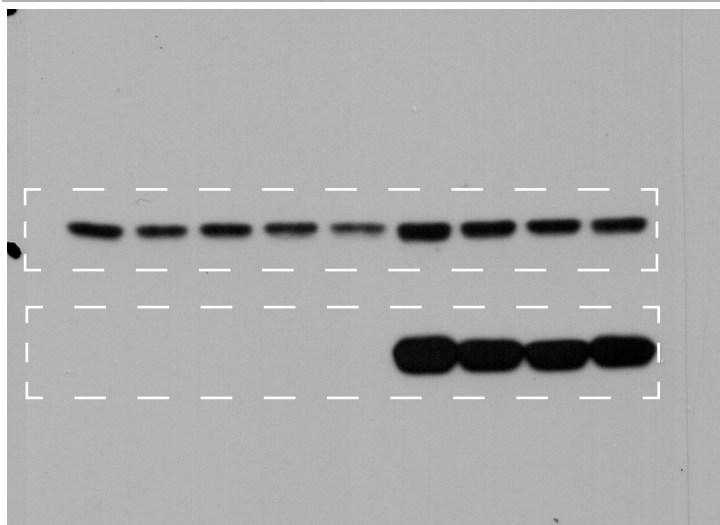

Supplement: Figure 8—source data 4. [file elife-97151-fig8-data4.pdf]

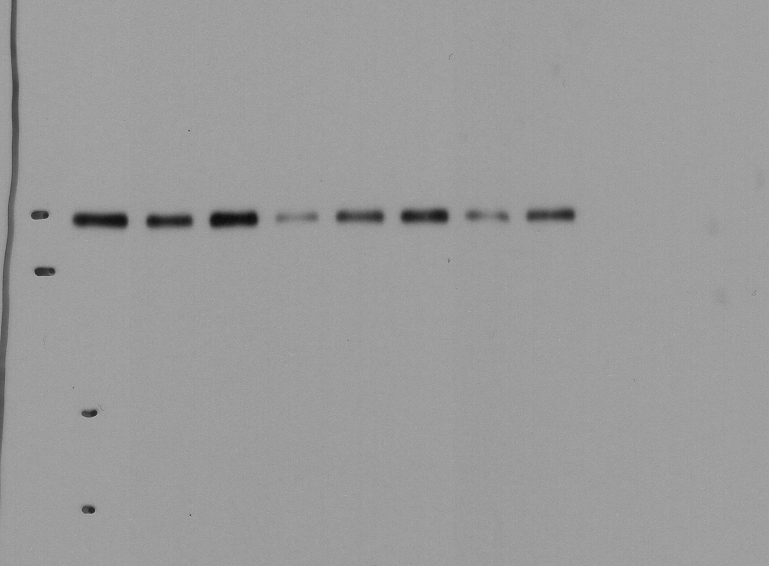

Supplement: Figure 8—figure supplement 1—source data 1. [file elife-97151-fig8-figsupp1-data1.zip › Fig S7A MAG ABE.tif]

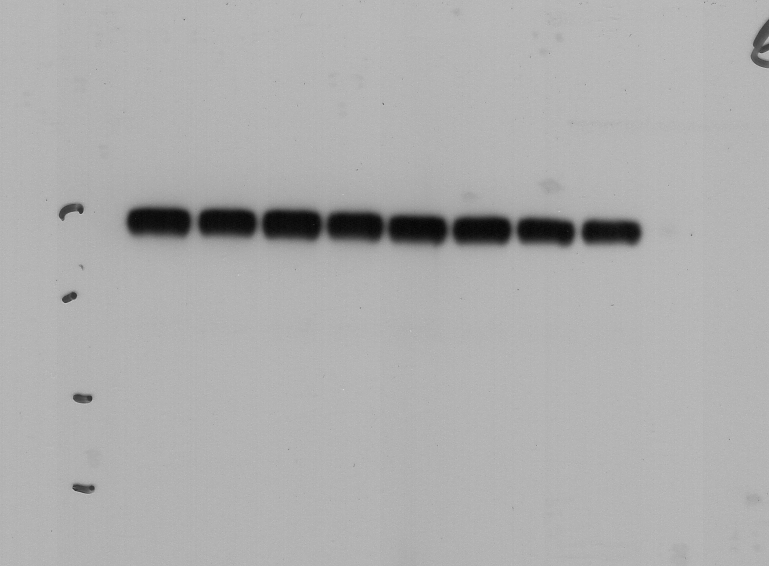

Supplement: Figure 8—figure supplement 1—source data 1. [file elife-97151-fig8-figsupp1-data1.zip › Fig S7A MAG Inputs.tif]

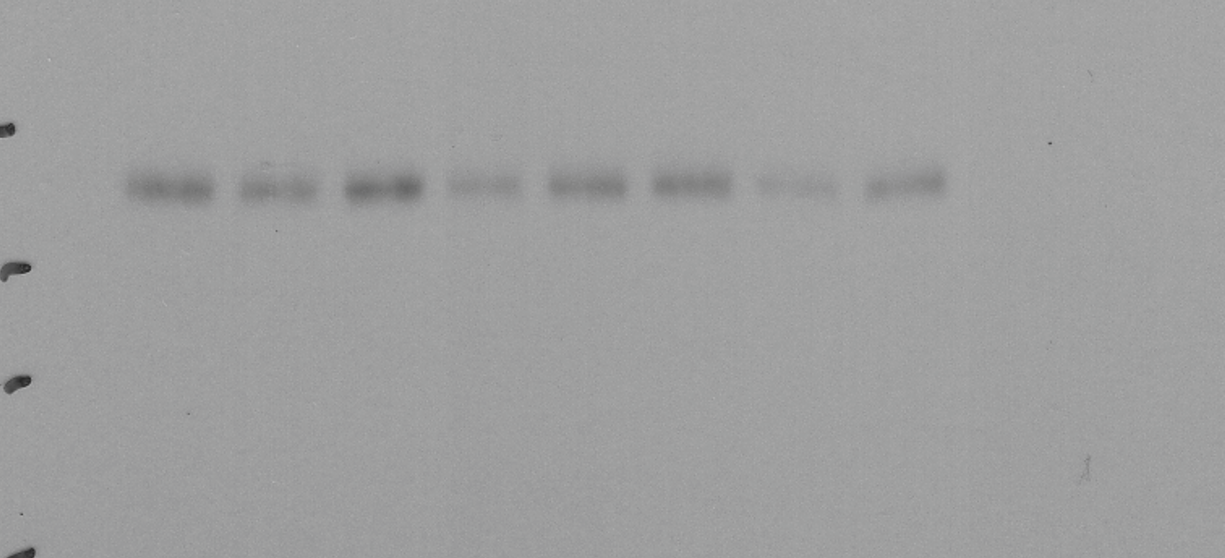

Supplement: Figure 8—figure supplement 1—source data 1. [file elife-97151-fig8-figsupp1-data1.zip › Fig S7C Cadm4 ABE.tif]

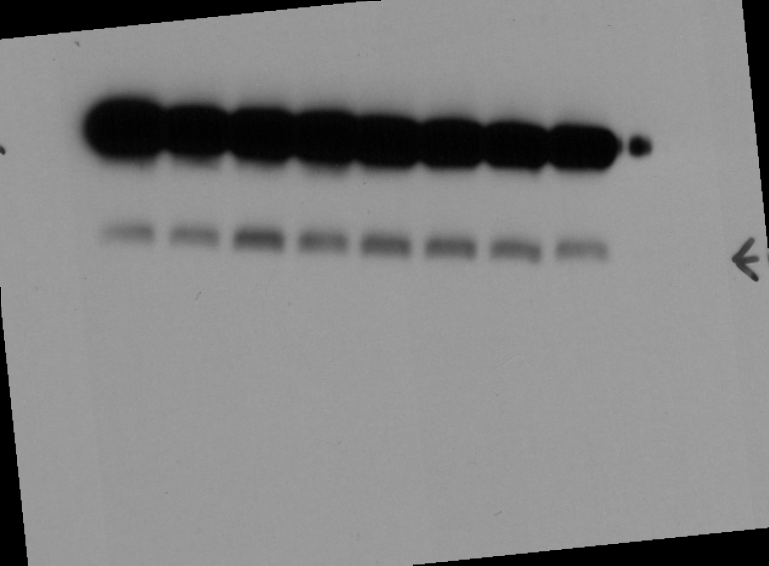

Supplement: Figure 8—figure supplement 1—source data 1. [file elife-97151-fig8-figsupp1-data1.zip › Fig S7C Cadm4 Inputs.tif]

wt wt wt wt Zdhhc9 KO  
Zdhhc9 KO  
Zdhhc9 KO  
Zdhhc9 KO  
wt (-NH<sub>2</sub>OH)

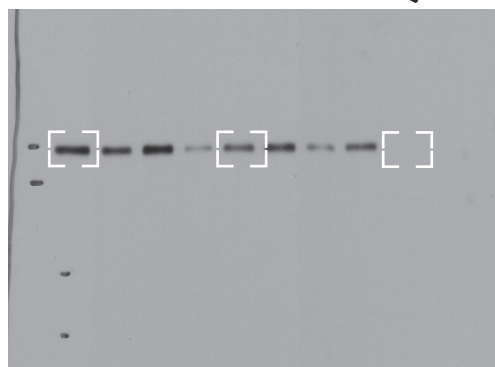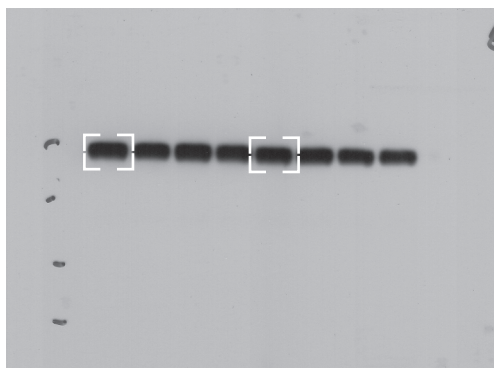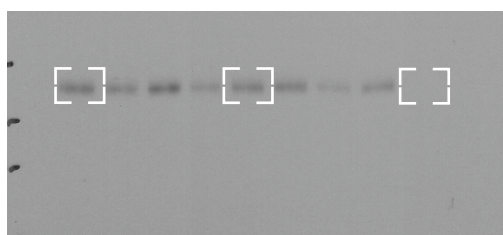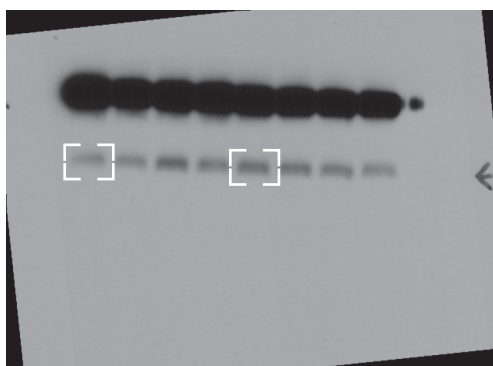

Supplement: Figure 8—figure supplement 1—source data 2. [file elife-97151-fig8-figsupp1-data2.pdf]
